# Downsizing food: a systematic review and meta-analysis examining the effect of reducing served food portion sizes on daily energy intake and body weight

**DOI:** 10.1017/S0007114522000903

**Published:** 2023-03-14

**Authors:** Eric Robinson, India McFarland-Lesser, Zina Patel, Andrew Jones

**Affiliations:** Department of Psychology, Eleanor Rathbone Building, University of Liverpool, Liverpool L69 7ZA, UK

**Keywords:** Portion size, Energy intake, Obesity, Food reformulation, Weight loss

## Abstract

Portion sizes of many foods have increased over time. However, the size of effect that reducing food portion sizes has on daily energy intake and body weight is less clear. We used a systematic review methodology to identify eligible articles that used an experimental design to manipulate portion size served to human participants and measured energy intake for a minimum of 1 d. Searches were conducted in September 2020 and again in October 2021. Fourteen eligible studies contributing eighty-five effects were included in the primary meta-analysis. There was a moderate-to-large reduction in daily energy intake when comparing smaller *v.* larger portions (Standardised Mean Difference (SMD) = –0·709 (95 % CI: –0·956, –0·461), approximately 235 kcal (983·24 kJ)). Larger reductions to portion size resulted in larger decreases in daily energy intake. There was evidence of a curvilinear relationship between portion size and daily energy intake; reductions to daily energy intake were markedly smaller when reducing portion size from very large portions. In a subset of studies that measured body weight (four studies contributing five comparisons), being served smaller *v.* larger portions was associated with less weight gain (0·58 kg). Reducing food portion sizes may be an effective population-level strategy to prevent weight gain.

Large portion sizes of commercially available food products have been identified as a likely contributor to the rise in overweight and obesity across the developed world^(1–3)^. Food portion sizes have increased over time, and the current food environment is characterised by a wide availability of energy-dense food products sold in larger portion sizes^(2,4–6)^. There is also now a consistent body of evidence indicating that manipulating the portion size of a meal served affects acute energy intake during that meal^(7–9)^. A meta-analysis of short-term studies estimated that doubling the served portion size at a meal increases acute meal energy intake by 35 %^(10)^. Based on these findings, public health measures to reduce portion sizes of food and drink products have been proposed as a potentially effective intervention to reduce obesity^(11)^.

The longer-term effects of reducing food portion sizes are less clear because reviews to date have only focused on the immediate effect that portion size has on acute energy intake at a single meal. Recent findings indicate that smaller portion sizes may ‘normalize’ overtime and be accepted by consumers^(12–14)^. However, less research has examined whether consumers ‘compensate’ for reduced portion sizes by eating more at later meals and whether reductions in portion size meaningfully affect daily energy intake and body weight^(5,15)^. For example, one laboratory study found decreasing the portion size of a main course served at lunch resulted in decreased energy intake from the main course, but resulted in an increase in the amount of energy consumed at dessert^(16)^. Lewis *et al.* ^(17)^ examined the effect of reducing breakfast portion size relative to a larger portion size served in the laboratory. However, there was no significant difference between portion size conditions in total daily energy intake, which included laboratory meals and participant self-reported intake outside of the laboratory^(17)^. Conversely, other studies measuring energy intake in the laboratory have found that serving smaller relative to larger served portion sizes resulted in lower daily energy intake over multiple days^(18,19)^. If smaller portion sizes do decrease daily energy intake, it is also unclear what the approximate size of this relationship is likely to be (i.e. changing portion size so that energy content of food served is decreased by 100 kcal (418·4 kJ) results in *x*kcal reduction in daily energy intake) and this is of particular importance to understanding the effect that portion size has on total diet.

At present, there is no consensus on the causal effect that manipulating portion size has on body weight. The dual intervention point model of energy balance and body weight proposes that environmental factors that increase or decrease energy intake (such as portion size) go largely uncompensated for (i.e. no significant counterbalancing via energy expenditure or long-term reductions in appetite) unless the amount of weight gained or lost is substantial and passes ‘intervention’ points at which some degree of physiological control causes compensatory responses that promote survival by preventing further weight gain or loss^(20)^. In a similar vein, the general model of intake regulation suggests that environmental factors that promote increased energy intake are largely uncompensated for and therefore can shift body weight upwards over time^(21)^. We therefore propose that if manipulating portion size does have an effect on daily energy intake that is maintained over several days, then changes to body weight would also be expected. To date, research examining the impact that portion size manipulations have on body weight has produced mixed findings, which may be due to studies lacking sufficient statistical power to detect relatively modest changes in body weight^(22–24)^. French *et al.* examined weight change in response to different portion sizes of takeaway lunches over a 6-month period^(22)^ and Jeffrey *et al.* examined weight change over a 4-week period in the real world^(24)^. Conversely, two other studies that used controlled laboratory procedures and manipulated portion size for multiple meals examined changes in weight over 4 and 5 d periods^(18,23)^. Although the latter two studies are relatively short in duration, changes in body weight have been observed as a result of increased daily energy intake for 3 d^(25)^. However, studies examining the effects of portion size on either daily energy intake or body weight outcomes are yet to be reviewed and meta-analysed.

Moving beyond existing systematic reviews of the impact that portion size has on acute single meal energy intake^(7,10)^, the aims of the present research were to systematically review and meta-analyse the impact that experimentally manipulating portion size has on total daily energy intake (as opposed to acute meal intake) and subsequent changes in body weight.

## Method

### Eligibility criteria and study selection

We included studies that used an experimental design to directly manipulate the portion size of food served to participants and measured energy intake across the course of at least 1 d.

#### Participants

Studies of human participants were eligible. Studies that sampled participants with a diagnosed medical/chronic health condition or currently undergoing treatment that may influence appetite (e.g. diabetes, bariatric surgery patients) were not eligible. There were no other exclusion criteria based on participant characteristics.

#### Intervention

Studies were required to have manipulated portion sizes (i.e. amount of food served to participants, also known as ‘serving size’ and characterised in the present review as kcal served) provided to participants. Studies that manipulated the portion size of a single food/meal were eligible, as were studies that manipulated all foods/meals served across the day. Studies that only reduced portion size of drink(s) were not eligible, as our focus was on food. However, if a study manipulated food and drinks, it was deemed eligible. Studies were required to serve or provide all participants with the same food type and to have achieved different portion size conditions by only altering the weight/volume of food served.

#### Intervention (smaller portion sizes) *v*. comparator (larger portion sizes) conditions

In studies with two portion size conditions, the ‘comparator’ condition was the larger portion size condition and the ‘intervention’ condition was the smaller portion size condition. Some studies described their manipulation as examining effect of ‘larger’ portion sizes *v.* ‘standard’ portion sizes on energy intake, and so to ensure consistency with the above conceptualisation, we treated the larger condition as the comparator condition. Studies with multiple portion size conditions (e.g. 100 % *v.* 75 % *v.* 50 %) were eligible and contributed multiple effects to the present review (e.g. 100 % *v.* 75 %, 75 % *v.* 50 %, 100 % *v.* 50 %).

#### Outcomes

Eligible studies were required to have measured energy intake across the course of a minimum of 1 d. Studies that measured energy intake through objective measurement (e.g. weighing of food pre/post eating), participant self-reported (e.g. dietary recall data) or a combination were eligible.

#### Study design

Studies that adopted a within-subjects/repeated-measures design (i.e. participants receive both smaller and larger portions) or between-subjects designs (i.e. participants were randomised to receive either the smaller *v.* larger portions), studies that measured energy intake in controlled laboratory or in real-world settings and studies that required participants to consume a meal or food in full *v.* not (e.g. compulsory consumption of a set amount of breakfast) were eligible. Studies that ‘crossed’ a portion size manipulation with another study manipulation (e.g. manipulation of both portion size and energy density of food served in the same study) were eligible, although only contrasts between portion size conditions were included. For studies that did not manipulate all meals/foods (e.g. only manipulating portion size of lunch), eligible studies were required to measure and report energy intake at that meal(s) that energy portion size was manipulated, in order to quantify the effect of the portion size manipulation independent of non-manipulated foods/meals.

### Article identification strategy

In September–October 2020, we searched PsycINFO, PubMed and SCOPUS (from date of inception onwards) using combinations of search terms relating to portion size and energy intake (see journal online supplementary materials text or https://osf.io/dj4yf/). To identify further published literature, we used a snowballing approach by searching the reference lists of eligible papers and by contacting authors to ask whether they had authored any other potentially eligible studies. To identify grey literature (to minimise publication bias), we conducted additional searches of the Open Science Framework (OSF) preprint archive (a database covering thirty other preprint archives, including PsychArxiv and Nutrixiv). Two authors independently screened and judged the eligibility of articles identified through electronic searches. A single author identified potentially eligible articles using the snowballing and grey literature approaches, and all potentially eligible articles were verified by a second independent author. Discrepancies for eligibility were resolved by discussion or were adjudicated by a third author. Searches were also re-run on 27 October 2021 to identify any new articles or preprints published, although no new eligible articles were identified.

### Data extraction

Two authors extracted the following information and any extraction discrepancies were resolved through discussion or a third author adjudicated; study sample information (e.g. country of study, participant group sampled, summary information on participant demographic characteristics and exclusion criteria for participant eligibility), portion size manipulation information (e.g. foods/meals manipulated, number of kcal served in portion size conditions and total number of kcal served/d in portion size conditions), study design information (e.g. within-subjects *v.* between-subjects design), measurement of energy intake (self-reported *v.* researcher measured), use of *ad libitum* intake *v.* compulsory intake (i.e. whether any meals were required to be eaten in full as part of the method), number of days energy intake was measured for, energy intake information (e.g. energy intake from portion size manipulated meals, non-manipulated meals and total daily energy intake and correlation between comparator *v.* intervention energy intake), results of any participant characteristic moderation analyses reported (e.g. does effect of portion size on energy intake differ in normal weight *v.* participant with obesity?), whether body weight was measured before and after each comparator *v.* intervention condition and risk of bias indices (see below).

### Risk of bias indicators

Informed by best practice guidelines for randomised control trials and experimental studies of eating behaviour^(26–29)^, studies were coded for nine risk of bias indicators that could vary between eligible studies. We opted for this approach rather than using a generic risk of bias tool (e.g. Cochrane) because existing tools omit key bias indicators relevant to portion size experiments. Studies that relied on self-reported energy intake (as opposed to researcher measured), did not use key participant exclusion eligibility criteria (e.g. use of medication affecting appetite, currently pregnant), were missing key methodological details, did not report use of random allocation to conditions, required participants to consume some meals/food in full, did not address demand characteristics (e.g. no attempt to blind participants to study aims or check if participants were aware of study aims), had a small sample size (N < 12 for within-subject studies), were not pre-registered, failed to report information on conflicts of interest statement (or reported a relevant conflict), were considered higher in risk of bias.

### Analyses

Pre-registered analyses and study data are available online: https://osf.io/dj4yf/. Authors were contacted and asked to provide details if statistical information required for analyses examining energy intake or body weight outcomes was missing. No within-subject/repeated-measures studies reported the correlation between daily energy intake in the larger *v.* smaller portion size conditions. We contacted all study authors to request this information and calculated the average (*r* = 0·8). As only a minority of authors provided this information, in sensitivity analyses, we examined if results of meta-analyses differed based on the correlation (including *r* = 0·4 and *r* = 0·6). Studies on portion size were initially intended to be part of a larger project that also included studies examining the effects of energy density on daily energy intake. However, prior to data extraction, the scope of the larger project was deemed too substantial and we therefore focus on portion size experiments in the current report. More detailed information and deviations from planned analyses are reported in the journal online supplemental material text.

### Primary analyses

#### Effect of portion size condition on daily energy intake

In a primary model, we examined the effect of portion size condition (smaller *v.* larger) on daily energy intake for all included studies. Because individual studies contributed multiple portion size comparisons, we used multi-level meta-analysis to account for the dependency of these effects^(30)^. We defined outliers as any effect sizes for which the upper bound of the 95 % CI was lower than the lower bound of the pooled effect CI (i.e., extremely small effects) or for which the lower bound of the 95 % CI was higher than the upper bound of the pooled effect CI (extremely large effects), using standardised effects. We identified influential cases as any effects with difference in beta values (DFBETA) values > 1 (indicative of a > 1 change in the standard deviation of the estimated coefficient after removal)^(31)^. We conducted Egger’s test^(32)^ and a trim and fill procedure^(33)^ to examine potential publication bias. See journal online supplementary materials for more detailed information. If we identified any outliers, they were removed in all subsequent primary analyses on daily energy intake to minimise their influence in meta-regression and subgroup analyses (although results with outliers included were similar). We calculated the standardised mean difference as a measure of effect size and Standardised Mean Difference (SMD) of 0·2, 0·5 and 0·8 are typically considered small, moderate and large-sized effects^(34)^. To aid interpretation, where appropriate, we also meta-analysed and presented the mean difference in energy intake (kcal) between portion size conditions. All analyses were conducted in R, using ‘metafor’ package.

#### Participant and study features: effects on daily energy intake

We conducted subgroup analyses to examine if results differed between effects drawn from female *v.* male samples and between studies that manipulated portion size at a majority of meals during the day (>2 meals) *v.* fewer meals (≤2 meals). We planned to examine other participant characteristics (e.g. normal weight *v.* overweight) in subgroup analyses but were unable to because too few studies reported sufficient data. Studies were variable in the number of days that they measured energy intake, and some studies reported effects on daily energy intake for each day of the study duration (effect of portion size on energy intake for days 1, 2, 3 etc.), so meta-regression was used to examine whether the impact of portion size on daily energy intake differed based on the number of days energy intake was assessed for. One study examined energy intake at a 6-month follow-up; as this was a much longer follow-up period compared with the other studies, we excluded this data point from the meta-regression (although results were consistent with its inclusion). We also used meta-regression to examine if the effect of portion size on daily energy intake was related to the % magnitude of portion size reduction (i.e. smaller portion being 50 % reduced compared with the larger portion) and difference in energy (kcal) served between the two portion size conditions. Variables examined in subgroup and meta-regressions were analysed independently.

#### Risk of bias indicators: effects on daily energy intake

We conducted subgroup analyses to examine if results from the primary analyses differed based on measurement of energy intake (researcher measured only *v.* use of self-report), whether studies required participants to consume any meals in full (yes *v.* no), use of random allocation to portion size conditions (yes *v.* no) and whether demand characteristics were addressed in the study (yes *v.* no).

### Secondary analyses

#### Compensation effects

A subset of studies did not manipulate portion size at every meal and reported energy intake during the manipulated and/or energy intake post-manipulated meal. In a series of analyses limited to these studies, we meta-analysed the effect of portion size on daily energy intake, manipulated meal energy intake and post-manipulated meal energy intake, to quantify the extent to which acute changes in energy intake caused by reducing portion size were later compensated for. In three studies, the manipulated meal was ‘fixed’ (i.e. eaten in full by all participants), resulting in a sd of 0. In sensitivity analyses, we imputed the standard deviations for this fixed meal as the average standard deviation (as a proportion of the mean) calculated from the non-fixed meals (approximately 29 %).

#### Curvilinear relationship

Previous research has suggested that there may be a curvilinear relationship between increases in portion size and energy intake^(10,35)^, whereby the effect that portion size has is smaller at larger more extreme portion sizes (e.g. medium *v.* large) compared with smaller portion sizes (e.g. small *v.* medium). A subset of studies (*n* 5) included three portion size conditions (e.g. large, medium and small) with similar-sized absolute increments in served portion size. We meta-analysed these studies and examined whether the reduction from the largest portion size (e.g. large *v.* medium contrast) produced a similar effect on daily energy intake as the same sized from reduction from the intermediate portion size (e.g. medium *v.* small) using subgroup analysis. Note: studies did not tailor portion sizes provided to participants based on individual energy needs, so ‘smaller’, ‘medium’ and ‘larger’ refer to size differences in each study.

#### Effect of portion size condition on body weight

For studies that also measured body weight change, we conducted a generic variance inverse meta-analysis on change in body weight (difference in change in body weight between the large and small portion size condition). If studies had more than two portion size conditions, because relatively subtle changes in energy intake would be unlikely to have a detectable effect on body weight over the short duration of studies included, to maximise statistical power, *a priori* we planned to include the smallest and largest portion size condition from each study.

## Results

### Study characteristics

**Fig. 1.**
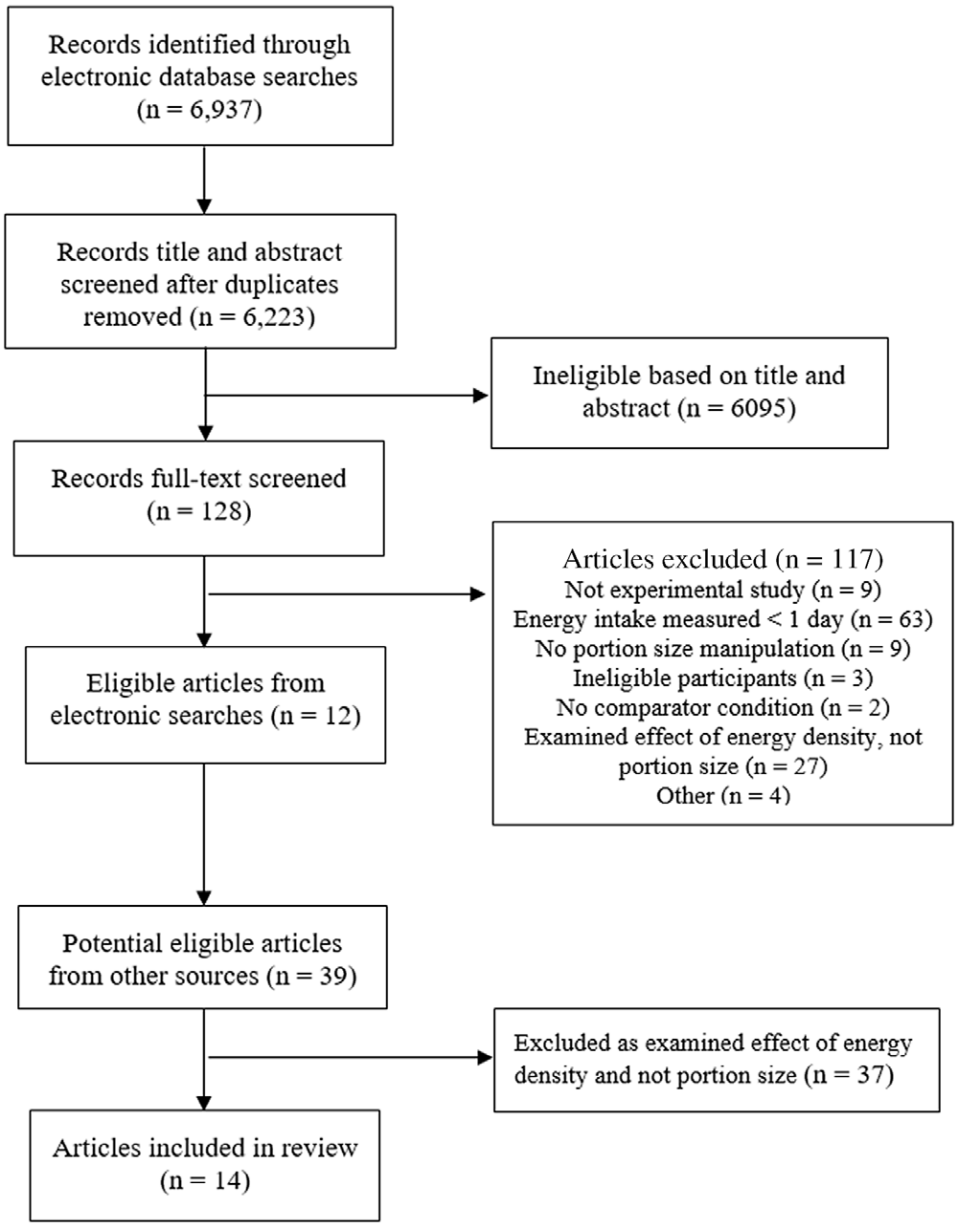
Study selection flowchart.

A total of fourteen studies were included in the review, see Fig. 1 for study selection flowchart. All studies were reported in published journal articles. Nine studies were from the USA, four were from the UK and one study was from Singapore. The majority of studies sampled from university staff/students and the local community (12/14). Nine studies sampled males and females, four sampled females only and one sampled males only. Twelve studies were in adults and two were in children. Of the thirteen studies that reported mean BMI, for nine studies mean BMI was within the normal BMI range (18·5–24·9) and four studies had a mean BMI above this range (BMI ≥ 25). Thirteen studies used within-subjects designs (portion size manipulated within participants) and one used a between-subjects design (portion size manipulated between participants). The total number of participants in each study ranged from *n* 19 to *n* 172. Portion size was manipulated and energy intake was measured for 1 d in six studies, between 2 and 11 d in six studies, in one study for 4 weeks and in another study for 6 months. Six studies manipulated portion size at a single meal and the remaining eight studies manipulated portion size at multiple meals. See Table 1 for individual study information.

**Table 1.** Summary information on included studies

Study	Country and sample	Sample characteristics	Study design information	Number of participants in analysis	Foods served	Portion size manipulation	Body weight measurement pre–post comparator and intervention conditions
Blatt 2012^(48)^	USA Local community and university	Men (*n* 28) Age: M = 26·8 years BMI: M = 24·9 kg/m^2^ Women (*n* 40) Age: M = 27·6 years BMI: M = 23·3 kg/m^2^	Within-subjects Researcher measured EI EI measured for 1 d	*n* 68 28M, 40F	PS of main entrée of all meals (breakfast, lunch and dinner) manipulated Sides and beverages not manipulated and consumed *ad libitum* Compulsory eating of manipulated entrees	Men Smaller PS condition 1000 kcal* served/d for manipulated meals 5337 kcal served/d kcal eaten/d: M = 2455, se = 97 Larger PS condition 1570 kcal served/d for manipulated meals 5904 kcal served/d kcal eaten/d: M = 2751, se = 92 Women Smaller PS condition 700 kcal served/d for manipulated meals 4146 kcal served/d kcal eaten/d: M = 1805, se = 57 Larger PS condition 1100 kcal served/d for manipulated meals 4549 kcal served/d kcal eaten/d: M = 1988, se = 58	Not measured
Fisher, 2007^(49)^	USA Local community (low-income family children)	Children Age: M = 5 years BMI: M = 60th percentile Sex: 24M, 35F Mothers (*n* 58) Age: M = 30 years BMI: M = 34 kg/m^2^	Within-subjects Researcher measured EI EI measured for 1 d	*n* 116	PS of main entrée of all meals (breakfast, lunch and dinner) manipulated and afternoon snack Sides and beverages not manipulated No compulsory eating, all foods consumed *ad libitum*	Children Smaller PS condition 2727 kcal served/d kcal eaten/d: M = 1500, sd = 359 Larger PS condition 4006 kcal served/d kcal eaten/d: M = 1639, sd = 378 Mothers Smaller PS condition 4109 kcal served/d kcal eaten/d: M = 2819, sd = 502 Larger PS condition 5974 kcal served/d kcal eaten/d: M = 2965, sd = 616	Not measured
French, 2014^(22)^	USA Large metropolitan medical complex employees	Age: M = 42·6 years BMI: M = 29·8 kg/m^2^ Sex: 60M, 112F	Between-subjects Self-report measured EI EI measured three times per week at months 1, 3 and 6	*n* 172	PS of lunch manipulated All other meals and snacks not manipulated (free-living) No compulsory eating	Small Lunch Box (approximately 400 kcal): 413 kcal served per manipulated meal (lunch) kcal eaten/d: M = 1718, se = 70 Medium Lunch Box (approximately 800 kcal): 821 kcal served per manipulated meal (lunch) kcal eaten/d: M = 1792, se = 68 Large Lunch Box (approximately 1600 kcal): 1604 kcal served per manipulated meal (lunch) kcal eaten/d: M = 1996, se = 71	Small lunch box M kg = −0·1, se = 0·43 Medium lunch box M kg = −0·1, se = 0·42 Larger lunch box M kg = 1·1, se = 0·44
Gray, 2002^(50)^	UK Students and staff at a University	Age: M = 24·3 years BMI: M = 22·6 kg/m^2^ Sex: 20M	Within-subjects Researcher and self-report measured EI EI measured for 1 d	*n* 20	PS of soup preload prior to lunch manipulated *Ad libitum* consumption of pasta test meal (lunch). All other meals and snacks not manipulated (free-living) Compulsory eating of soup preload	Smaller PS condition 100 kcal served per manipulated meal (soup) kcal eaten/d: M = 2756·00, se = 158·9 Larger PS condition 301 kcal served per manipulated meal (soup) kcal eaten/d: M = 2798·40, se = 155	Not measured
Haynes, 2020^(18)^	UK Local community and university	Age: M = 31·6 years BMI: M = 26·0 kg/m^2^ 37 % normal weight 63 % overweight/obesity Sex: 15M, 15F	Within-subjects Researcher measured EI EI measured for 5 d	*n* 30	PS of lunch and dinner entrée manipulated *Ad libitum* eating of all other meals/sides/snacks No compulsory eating	Smaller than normal PS condition: 678 kcal served/d for manipulated meals (lunch and dinner entrees) 5074 kcal served/d kcal eaten/d: M = 2238, sd = 490 Small-normal PS condition: 1086 kcal served/d for manipulated meals (lunch and dinner entrees) 5485 kcal served/d kcal eaten/d: M = 2448, sd = 584 Large-normal PS condition: 1494 kcal served/d for manipulated meals (lunch and dinner entrees) 5897 kcal served/d kcal eaten/d: M = 2543, sd = 592	Smaller than normal PS condition M kg = −0·03, sd = 1·55 Small-normal PS condition M kg = −0·03, sd = 1·34 Large-normal PS condition M kg = 0·33, sd = 0·74
Jeffery, 2007^(24)^	USA Local community and university	Age: M = 33·0 years BMI: M = 28·9 kg/m^2^ Sex: 19F	Within-subjects Self-report measured EI EI measured two times per week for 4 weeks	*n* 19	PS of lunch manipulated *Ad libitum* eating of lunch. All other meals and snacks not manipulated (free-living) No compulsory eating	Smaller PS condition 767 kcal served per meal kcal eaten/d: M = 1875, sd = missing Larger PS condition 1528 kcal served per meal kcal eaten/d: M = 2153, sd = missing	Smaller PS condition M kg = 0·06, sd = 1·03 Larger PS condition M kg = 0·64, sd = 1·16
Kelly, 2009^(23)^	UK Local community and university	Men (*n* 21) Age: M = 29·7 years BMI: M = 25·3 kg/m^2^ 43 % normal weight 57 % overweight/obesity Women (*n* 22) Age: M = 31·7 years BMI: M = 23·7 kg/m^2^ 68 % normal weight 32 % overweight/obesity	Within-subjects Researcher measured EI EI measured for 4 d	*n* 43	PS of all meals, snacks and drinks manipulated *Ad libitum* eating of all food and drink No compulsory eating	Men Smaller PS condition kcal served/d/meal - not reported kcal eaten/d: M = 3490, se = 220 Larger PS condition kcal served/d/meal - not reported kcal eaten/d: M = 4056, se = 239 Women Smaller PS condition kcal served/d/meal - not reported kcal eaten/d: M = 2721, se = 137 Larger PS condition kcal served/d/meal - not reported kcal eaten/d: M = 2995, se = 137	Men: Smaller PS condition M kg = 0·1, sd = missing (imputed as 1·2) Larger PS condition M kg = 0·9, sd = missing (imputed as 1·2) Women: Smaller PS condition M kg = 0·2, sd = missing (imputed as 1·2) Larger PS condition M kg = 0·6, sd = missing (imputed as 1·2)
Kral, 2004^(51)^	USA Local community and university	Age: M = 23·4 years BMI: M = 23·1 kg/m^2^ Sex: 39F	Within-subjects Researcher measured EI EI measured for 1 d	*n* 39	PS of entrée at lunch manipulated All foods and beverages were consumed *ad libitum* except for compulsory food items with lunch Compulsory eating of side dishes at lunch	500 g condition: 750 kcal served per manipulated meal (lunch entree) kcal eaten/d: M = 1745, se = 57 700 g condition: 1050 kcal served per manipulated meal (lunch entrée) kcal eaten/d: M = 1782, se = 51 900 g condition: 1350 kcal served per manipulated meal (lunch entrée) kcal eaten/d: M = 1855, se = 54	Not measured
Lewis, 2015^(17)^	UK Local community and university	Age: M = 42·5 years BMI: M = 29·0 kg/m^2^ Adults with overweight and obesity only Sex: 15M/18F	Within-subjects Researcher and participant measured EI EI measured for 1 d	*n* 33	PS of breakfast meal manipulated *Ad libitum* eating of lunch and afternoon snack. All other meals and snacks not manipulated (free-living) Compulsory eating of breakfast	40 % reduction condition: 420 kcal served per manipulated meal (breakfast) kcal eaten/d: M = 2190, se = 104 20 % reduction condition: 559 kcal served per manipulated meal (breakfast) kcal eaten/d: M = 2365, sem = 117 ‘Control’ no reduction condition: 699 kcal served per manipulated meal (breakfast) kcal eaten/d: M = 2459, se = 94	Not measured
McCrickerd, 2017^(52)^	Singapore Local community and university	Age: M = 25·4 years BMI: M = 21·1 kg/m^2^ Sex: 53F	Within-subjects Researcher and self-report measured EI EI measured for 1 d	*n* 51	PS of breakfast meal manipulated *Ad libitum* eating of all meals All other meals and snacks not manipulated (free-living) No compulsory eating of meals	Smaller PS condition 399 kcal served per manipulated meal (breakfast) kcal eaten/d: M = 1668 kcal, sd = 521 Larger PS condition 599 kcal served per manipulated meal (breakfast) kcal eaten/d: M = 1689 kcal, sd = 489	Not measured
Rolls 2006a ‘Larger’^(53)^	USA Local community & university	Men (*n* 16) Age: M = 24·4 years BMI: M = 24·7 kg/m^2^ Women (*n* 16) Age: M = 21·2 years BMI: M = 22·2 kg/m^2^	Within-subjects Researcher measured EI EI measured for 2 d	*n* 32 (16M, 16F)	PS of for all meals and snacks manipulated *Ad libitum* eating of all meals and beverages Beverages were not manipulated No compulsory eating of meals	Men kcal served/d - not reported/calculable kcal eaten/d: 100 % condition: M = 2964 kcal, se = 133 150 % condition: M = 3461 kcal, se = 137 200 % condition: M = 3774 kcal, se = 198 Women kcal served/d - not reported/calculable kcal eaten/d: 100 % condition: M = 2188 kcal, se = 99 150 % condition: M = 2523 kcal, se = 99 200 % condition: M = 2717 kcal, se = 131	Not measured
Rolls 2006b ‘Reductions’^(54)^	USA Local community and university	Age: M = 21·9 years BMI: M = 22·6 kg/m^2^ Sex: 24F	Within-subjects Researcher measured EI EI measured for 2 d	*n* 24	PS of all food manipulated *Ad libitum* eating of all meals and beverages Beverages were not manipulated No compulsory eating of meals	Smaller PS condition 2846 kcal served/d kcal eaten/d: M = 1951, se = 65 Larger PS condition 3794 kcal served/d kcal eaten/d: M = 2207, se = 67	Not measured
Rolls, 2007^(19)^	USA Local community and university	Men (*n* 13): Age: M = 24·7 years BMI: M = 24·6 kg/m^2^ Women (*n* 10) Age: M = 25·8 years BMI: M = 22·9 kg/m^2^	Within-subjects Researcher measured EI EI measured for 11 d	*n* 23	PS of all meals (breakfast, lunch and dinner) and snacks manipulated *Ad libitum* consumption of all meals (breakfast, lunch and dinner) and snacks) No compulsory eating	Men: Smaller PS condition 4100 kcal served/d kcal eaten/d: M = 2909, se = 106 Larger PS condition 6150 kcal served/d kcal eaten/d: M = 3328, se = 114 Women: Smaller PS condition 3400 kcal served/d kcal eaten/d: M = 2073, se = 97 Larger PS condition 5100 kcal served/d kcal eaten/d: M = 2530, se = 79	Not measured
Smethers, 2019^(55)^	USA Children from childcare centres	Age: M = 4·4 years BMI: BMI-for-age percentile = 52·8 89 % normal weight 11 % overweight/obesity Sex: 30M, 16F	Within-subjects Researcher measured EI EI measured for 5 d	*n* 46	PS of all food and beverages manipulated *Ad libitum* eating of all meals and beverages No compulsory eating of meals	Smaller PS condition 1627 kcal served/d kcal eaten/d: M = 914, se = 44 Larger PS condition 2450 kcal served/d kcal eaten/d: M = 1081, se = 44	Not measured

EI, energy intake; M, mean; PS, portion size.

*Use the formula 1 kcal = 4·184 kJ to get the energy value in kJ.

From the fourteen studies, there were a total of eighty-five smaller *v.* larger portion size daily energy intake comparisons, thirty-five of which were from female-only samples, twenty-three male only and twenty-seven were mixed sex. The size of portion size reduction examined (energy served in a larger portion condition *v.* smaller portion condition) ranged from 20 % to 74 %, with a median of 33 %. For the sixty-five portion size comparisons that the difference in energy content (kcal) served between larger and smaller portion conditions was reported or calculable, the range of difference was 14 kcal/59 kJ (portion size of a single meal manipulated) to 1865 kcal/7803 kJ (all meals manipulated), with a median of 823 kcal/3443 kJ.

### Risk of bias

Only a minority of studies measured daily energy intake from participant self-reports as opposed to objective researcher measured energy intake (5/14). In a limited number of studies, participants were required to consume one or more meals in full (4/14) and few studies failed to address demand characteristics (4/14). Most studies reported no relevant conflicts of interest (9/14) and most studies (1/14) were not pre-registered (e.g. inclusion of a detailed analysis protocol). It was rare for studies to not report on key methodological information (2/14) or fail to report or use random allocation to conditions (5/14). No studies had small sample sizes and no studies failed to use key participant eligibility criteria (e.g. currently taking appetite affecting medication). See journal online supplementary material table S1 for individual study risk of bias information.

### Effect of portion size condition on daily energy intake

**Fig. 2.**
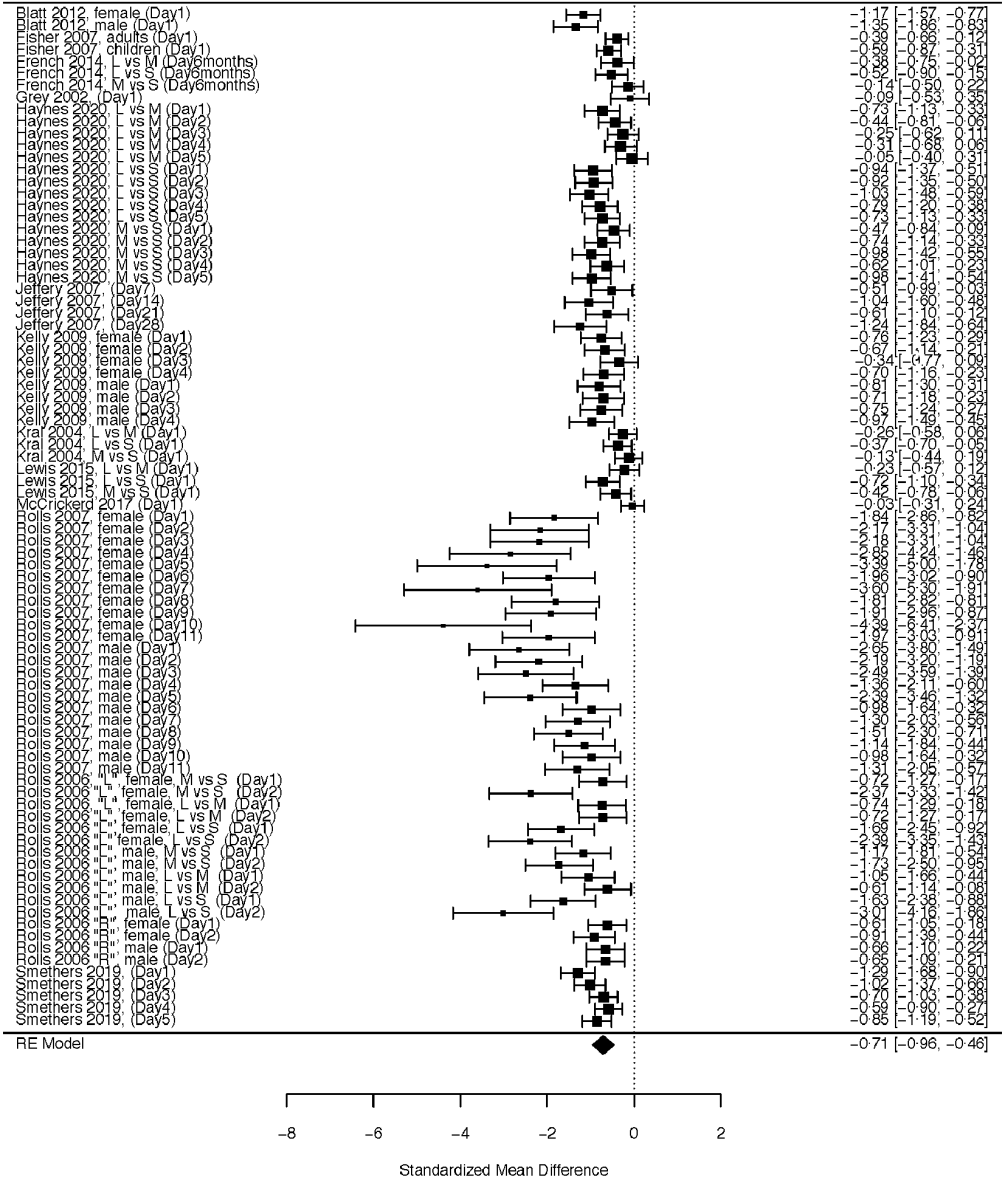
Primary meta-analysis of standardised mean difference in daily energy intake between small and large portion size conditions. L, M and S refer to the large, medium and small portion size conditions in a study.

Eighty-five effects from fourteen studies were included in the primary meta-analysis. The multi-level meta-analysis was a better fit for the data than a standard analysis (Loglikelihood ratio = 58·75, *P* < 0·001). There was a moderate-to-large reduction in daily energy intake, for smaller *v*. larger portions (SMD = –0·709 (95 % CI: –0·956, –0·461), Z = 5·62, *P* < 0·001, I^2^ = 80·6 %). See Fig. 2. Sensitivity analyses (i.e. varying within-subjects correlation) did not substantially influence the effect magnitude (SMD > 0·624) or statistical significance of the primary meta-analysis. Trim and fill imputed 25 effect sizes in a single-level model, which did not substantially influence the effect size (SMD = –0·667), and Egger’s test was significant indicative of bias (z = –14·08, *P* < 0·001), see journal online supplementary materials Fig. S1 for funnel plot. When removing thirteen outlying effect sizes in which the CI did not overlap with the pooled estimates (upper bound CI < –1·03; SMDs ranged from –2·17 to –4·39), the effect size remained moderate-to-large with a small reduction in heterogeneity (SMD = –0·660 (95 % CI: –0·860, –0·459), z = 6·43, *P* < 0·001, I^2^ = 74·9 %). For meta-analysed mean difference in daily energy intake expressed as kcal, smaller portions were associated with a reduction of –235·75 (–303·02 to –168·48) kcal (–986·378 kJ) consumed/d compared with larger portions. Removal of the outlying effects did not substantially reduce this (–221·86 (95 % CI: –275·69, –168·02)). See journal online Supplementary Materials Figures S2 and S3.

### Participant and study features: effects on daily energy intake

#### Impact of portion size on energy intake over time

We meta-regressed the day of assessment (range day: 1–28, mean = 3·98, median = 2) against the effect of portion size on energy intake, and there was no significant association (coefficient = –0·011 (95 % CI: –0·038, 0·016), Z = 0·81 *P* = 0·415), indicating that the influence portion size had on energy intake was not dependent on how long studies measured daily energy intake for.

#### The effect of manipulating most meals during the day *v.* fewer meals

There was a significant moderation effect (X^2^(1) = 10·24, *P* = 0·001). For studies in which two or fewer meals were served as smaller *v.* larger portions (30 effect sizes across 7 studies), the effect size was small-to-moderate (SMD = –0·429 (95 % CI: –0·622, –0·228), Z = 4·23, *P* < 0·001) and the change in kcal was −168·23 (–233·86 to −103·61). For studies in which more than two meals were served as smaller *v.* larger portion sizes (forty-two effect sizes across seven studies), there was a moderate-to-large effect size (SMD = –0·900 (95 % CI: −1·132, –0·669), Z = 7·63, *P* < 0·001) and the change in energy intake (kcal) was −268·53 (–1123·52 kJ) (–335·62, −201·44).

### Magnitude of portion size reductions

#### Reduction of portion size (percentage)

In meta-regression, the standardised effect size was negatively associated with the magnitude portion size reduction as a percentage (coefficient = –0·016 (95 % CI: –0·022, –0·009), Z = 4·62, *P* < 0·001), whereby based on the included studies a reduction of portion sizes served by 10 % was associated with a 1·6 % reduction in daily energy intake.

#### Reduction of portion size (kcal)

**Fig. 3.**
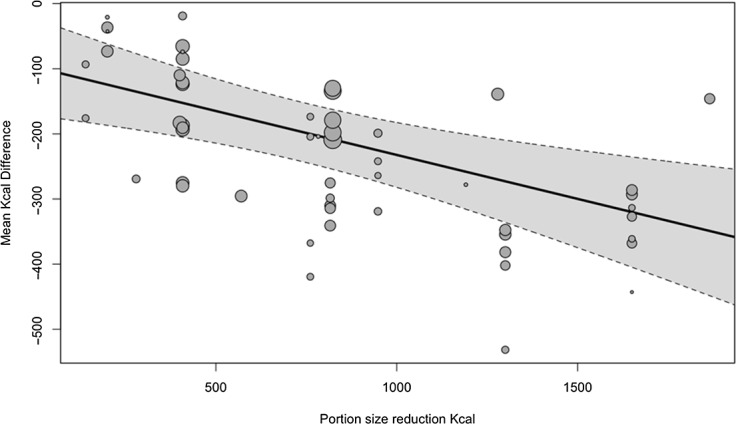
Association between the difference in kcal served by portion size conditions (x axis) and daily energy intake (y axis) change in kcal based on portion size reduction in kcal.

In meta-regression, the magnitude of portion size reduction expressed as a kcal difference between portion size conditions was negatively associated with total daily energy intake, coefficient = −0·135 (95 % CI: −0·214, −0·056), Z = 3·56, *P* < 0·001), whereby a 100 kcal total reduction in food portion size served was associated with a 14-kcal (58·57 kJ) reduction in daily energy intake. See Fig. 3.

### Risk of bias indicators: effects on daily energy intake

Whether energy intake was objectively measured (by the researcher) *v.* self-report methods moderated the effect of portion size on daily energy intake (X^2^(1) = 4·97, *P* = 0·026). The effect of portion size on daily energy intake for studies using researcher measured energy intake (sixty effect sizes from nine studies) was SMD = –0·804 ((95 % CI: −1·033, −0·575), Z = 6·87, *P* < 0·001), and for self-reported energy intake (twelve effect sizes from five studies) was SMD = –0·374 ((95 % CI: –0·640, –0·108), Z = 2·76, *P* = 0·00). Whether or not studies reported the use of random allocation to portion size conditions did not significantly affect results (X^2^(1) = 0·02, *P* = 0·884). Whether or not a study addressed demand characteristics (X^2^(1) = 0·03, *P* = 0·867) or required participants to consume any meals in full *v. ad libitum* (X^2^(1) = 0·71, *P* = 0·845) did not significantly affect results.

### Evidence for post-portion size manipulation compensatory effects

For fifteen effect sizes across seven studies that did not manipulate portion size for all meals, the impact of portion size on meal energy intake (at manipulated portion size meals) and later energy intake (at non-manipulated meals) were measured and reported separately. During the manipulated meal, there was a large-sized reduction for these fifteen effects (SMD = −1·60 (95 % CI: −2·362, −0·841), Z = 4·13, *P* < 0·001), and manipulated meal energy intake (expressed in kcal) was −232·92 (95 % CI: −357·64, −108·21), Z = 3·66, *P* < 0·001) when comparing smaller *v.* larger portion sizes. For non-manipulated meals following the portion size manipulated meals, there was a small-to-moderate sized increase in energy intake (kcal) after the meal in the smaller portion *v*. larger portion (SMD = 0·369 (95 % CI: 0·024, 0·714), Z = 2·10, *P* = 0·036, I^2^ = 70·5 %) and expressed as kcal the effect was 97·72 ((95 % CI 12·60, 182·83)). Note, the standardised effect was slightly smaller in sensitivity analyses (SMD = 0·226 (95 % CI: 0·010, 0·442), Z = 2·05, *P* = 0·040). Thus, changes to energy intake at meals caused by serving smaller portion sizes were in part later compensated for; approximately 42 % of the reduction in energy intake observed at manipulated portion size meals was ‘compensated for’ through additional energy intake at other meals later that day.

### Curvilinear relationship

**Fig. 4.**
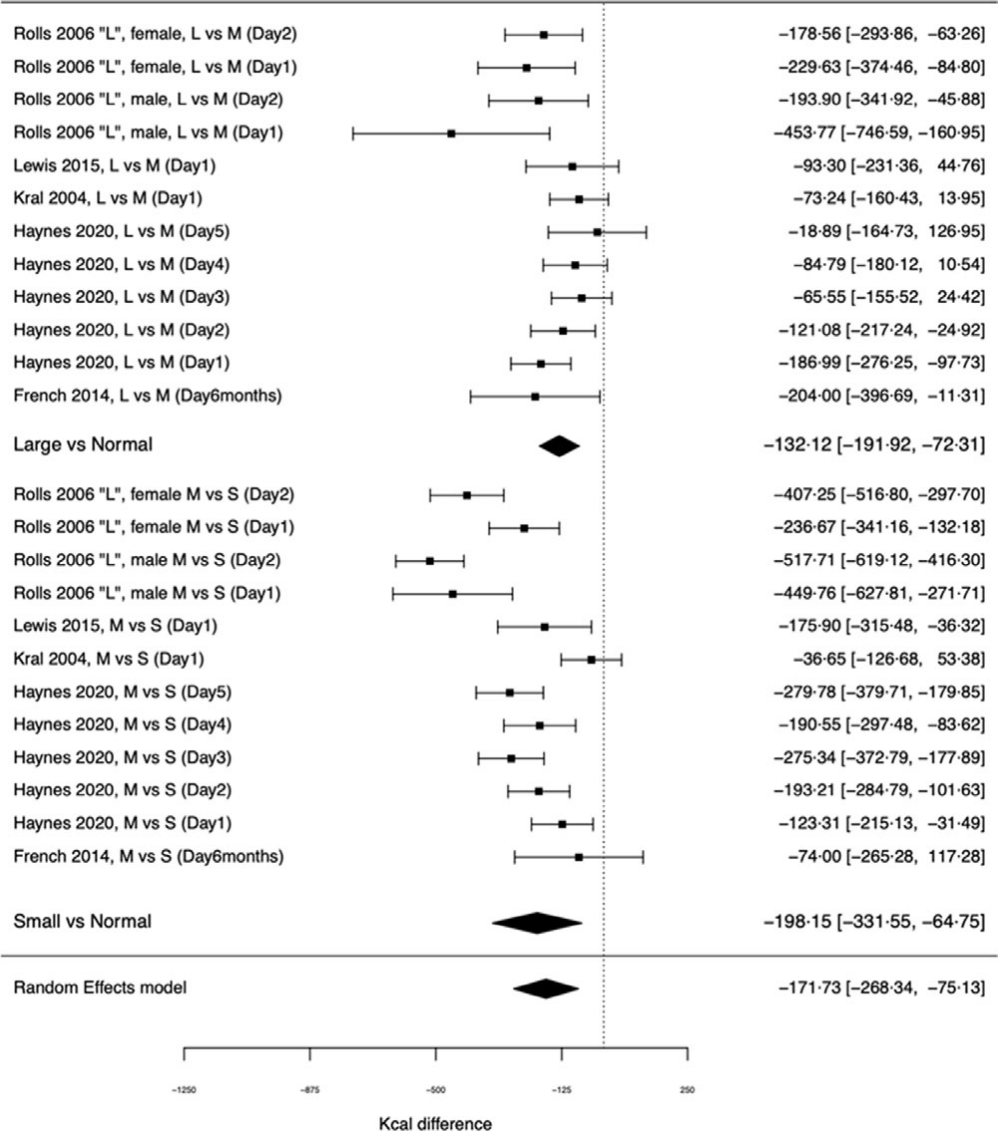
Effect of portion size on daily energy intake in studies allowing for examination of a curvilinear relationship. L, M and S refer to the large, medium and small portion size conditions in a study.

Examining the difference in the portion size effect between large *v*. normal (intermediate) portions and small *v*. normal (intermediate) portions demonstrated a significant moderation effect (X^2^(1) = 7·57, *P* = 0·006). In large *v*. normal portion comparison (twelve effect sizes across the five studies), the effect of portion size on daily energy intake was small-to-moderate in statistical size (SMD = –0·389 (95 % CI: –0·554, –0·224), Z = 4·61, *P* < 0·001), with a daily energy intake difference of –132·12 kcal (–552·79 kJ) (95 % CI: –191·92, –72·31). In small *v*. normal size portion comparisons, the effect was larger (SMD = –0·578 (95 % CI: –1·047, –0·109), Z = 2·43, *P* = 0·016), with a daily energy intake difference of –198·15 kcal (–829·05 kJ) (95 % CI: –331·55, –64·75). See Fig. 4 for kcal forest plot. Therefore, the impact that manipulating portion size has on daily energy intake is dependent on the size of portion that is decreased; decreasing portion size from the largest portions had a 33 % smaller impact on daily energy intake than decreasing portion size of medium (intermediate) portions.

### Effect of portion size condition on body weight

**Fig. 5.**
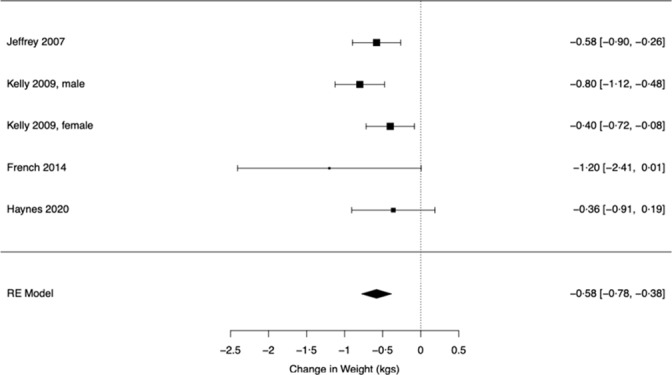
Effect of portion size condition on change in body weight.

Four studies (contributing five effects sizes) examined change in body weight in smaller *v.* larger portion size conditions. Portion sizes of one meal were manipulated in two of the studies, two meals were manipulated in one study and in the remaining study, all meals were manipulated. Study durations were 4 d, 5 d, 4 weeks and 6 months in duration and are described in detail in Table 1. The standardised effect of portion size on change in body weight was SMD = 0·536 ((95 % CI: 0·268, 0·803), Z = 3·92, *P* < 0·001, I^2^ = 47·0 %). The difference in change in kilograms was 0·579 (95 % CI: 0·384, 0·776), indicating that after allocation to being served smaller portions, participants gained 0·6 kg less weight than when served larger portions. See Fig. 5 for kg forest plot.

## Discussion

We systematically reviewed and meta-analysed studies that examined the effect of experimentally manipulating food portion sizes on daily energy intake. Across fourteen eligible studies, smaller food portions resulted in lower daily energy intake and this effect was consistent across males and females. Studies varied in duration from 1 d to 6 months, and there was no evidence that the effect of portion size on energy intake differed between studies that were shorter in duration or examined energy intake for longer. Reductions to daily energy intake were larger in studies that manipulated the portion size of foods at most meals as opposed to studies that only manipulated portion size at one or two meals. This pattern of results is likely to be explained by the finding that larger reductions to served food portion sizes (expressed as difference in total kcal served) resulted in larger changes to daily energy intake.

Meta-analyses of the effect of portion size on energy intake have been limited to studies measuring energy intake at a single acute meal, to date. In a meta-analysis of studies sampling children, larger (*v.* smaller) portion sizes were estimated to have a moderate-sized statistical effect on energy intake (SMD = 0·47)^(7)^. In a meta-analysis consisting of adults and children^(10)^, increasing portion size by 100 % resulted in on average a 35 % increase in meal energy intake (or in reverse a 50 % portion size reduction associated with a 19 % decrease in meal energy intake). An important contribution of the present analyses is that they move beyond existing reviews by addressing how acute effects of portion size may be compensated for over longer periods of time. After accounting for potential publication bias, the effect of decreasing portion size on daily energy intake in the present meta-analysis was a statistically moderate-sized effect. Based on the results of analyses of all included studies, a 50 % reduction in portion sizes would be associated with an 8 % decrease in daily energy intake, or expressed as energy; a 100 kcal/418 kJ total reduction to the energy content of portion sizes served would be estimated to result in approximately 14 kcal/59 kJ decrease to daily energy intake. Therefore, the longer-term effect of manipulating portion size on daily energy intake tends to be markedly smaller than when examining energy intake at a single meal. This observation is also likely to have relevance to other types of interventions designed to reduce energy intake and highlights the need to study energy intake beyond a single acute meal.

Consistent with some short-term studies^(10,35)^, we found evidence from a small subset of studies (*n* 5) that the effect of portion size on daily energy intake was curvilinear; reductions to daily energy intake were markedly smaller (approximately 33 %) when reducing portion size from a large portions to a ‘normal’/intermediate portion, compared with reducing portion size from a ‘normal’/intermediate portion. This is important because studies did not tailor portion sizes to energy needs of individual participants and most studies included served participants very large amounts of food in the ‘large’ portion size condition and these portions are unlikely to be representative of portion sizes served in everyday life. For example, results from a laboratory study^(19)^ examining the effect of very large portions (i.e. serving participants in excess of 6000 kcal/25104 kJ/d) found that a 2050 kcal/8577 kJ difference in energy served per day between the larger and smaller portion conditions of the study resulted only in a 419 kcal/1753 kJ difference in average daily energy intake (80 % ‘compensation’ in energy consumed compared with difference in energy served between conditions). Conversely in a different laboratory study that compared meals that were chosen to be perceived as being ‘normal’ in size (i.e. perceived as being typical of everyday portion sizes by participants) *v.* smaller portioned meals^(18)^, a 408 kcal/1707 kJ difference in energy served across the day resulted in far less ‘compensation’; a 210 kcal/879 kJ decrease in average daily energy intake (only 49 % compensation). We assume that curvilinear relations may be explained by stomach capacity. Consistent with the boundary model of food consumption^(36,37)^, there is likely to be a ‘biological zone of indifference’ for moderate-sized portions whereby a person can easily consume more energy without any obvious physiological consequences. However, there is of course a limit to the volume of food one can eat without experiencing discomfort even if trying to avoid food going to waste as a result of being served a very large portion^(38)^, resulting in further increases to large portion sizes having a reduced impact on energy intake. Irrespective of the exact cause of the curvilinear relationship portion size has with energy intake, curvilinear relations should be accounted for when extrapolating the results of our main analyses to estimate how much reducing portion sizes in everyday life would be expected to decrease daily energy intake.

Studies tended to be relatively low in risk of bias, and there was minimal evidence that studies higher in risk of bias (e.g. did not report use of random allocation to portion size conditions) produced different results to studies not exhibiting risk of bias. However, studies that relied in part on participant self-reports of food consumed to calculate energy intake reported smaller effects of portion size on daily energy intake than studies relying on researcher-measured energy intake. Given that participant self-reported energy intake is prone to recall bias and inaccuracy^(39)^, participant reporting biases may underestimate the effect of portion size on energy intake in some studies. We identified a relatively high number of outliers and this may reflect studies with more extreme portion size manipulations as opposed to erroneous results on daily energy intake. Although results were consistent in analyses, when outliers were *v.* were not excluded.

We propose that portion size impacts on daily energy intake because there appears to be a lack of tight short-term control of energy intake in humans^(40)^ and food intake behaviour is context-dependent, whereby individuals can easily eat more or less food dependent on the absence *v.* presence of environmental cues or factors, such as portion size. Consistent with other studies^(16)^, we found evidence that there is some energy intake compensation in response to manipulations of portion size (e.g. eating more/less after having been served a smaller/larger portion size), but this compensation was only partial and this compensation does not become larger over time. That compensation in response to smaller *v.* larger portions occurs each day but does not become larger over time may be explained by the short-term physiological regulation of food intake being determined by the emptiness of the gut and stomach^(40)^ (i.e. why smaller portions may promote some short-term increase in energy intake on the same day). Furthermore, because cognitive regulation of food intake is episodic memory specific and therefore influenced only by recent eating episodes^(41,42)^ (i.e. during the same day), any compensatory effects caused by perceived undereating would be expected to occur over relatively short time frames. However, consistent with the dual intervention point model, over longer periods any further physiological compensatory responses to decreased/increased energy intake caused by smaller/larger portion sizes would be predicted to only occur as a result of a substantial amount of weight loss or gain^(20)^.

Because portion sizes of some commercially provided foods have increased in recent times^(2)^, the present findings suggest that this is likely to have contributed to increases in population-level energy intake and the prevalence of obesity. There have been some questions raised over the lack of causal evidence on the effect of portion size on body weight and therefore the public health benefit of reducing portion sizes^(5)^. We meta-analysed a small subset of studies that measured participant body weight and found that larger portions were associated with greater weight gain than smaller portions. However, two of the studies were relatively short in duration (4–5 d) and conclusions are based on a limited number of eligible studies (four studies contributing five effect sizes to meta-analysis). Assuming that changes to energy intake caused by portion size manipulation are not physiologically compensated for over longer periods of time, the relatively short-term nature of the included studies will likely underestimate the effect of portion size on body weight. It would therefore be preferable for studies to measure the effect of manipulated portion size on body weight over longer time frames. Further replication of these findings will also be important as they suggest that reductions to food portion sizes may prevent population-level weight gain and therefore address prevalence of overweight and obesity.

We were limited to examining only sex as a moderating participant characteristic of the effect of portion size on daily energy intake and found no evidence of moderation. However, this subgroup analysis consisted of a small number of effects and therefore should be interpreted with caution. We were unable to examine whether participant BMI moderated the effect of portion size on daily energy intake, or the potential moderating effect of individual differences in trait eating behaviours, such as satiety responsiveness^(43)^. In addition, studies tended to sample university staff and students. Studies to date have not found convincing evidence for participant characteristics that consistently moderate the effect of portion size on energy intake^(44)^. However, it will be important for future research to address this and examine if the impact that reducing portion size has on daily energy intake is beneficial to the majority of the population. A further limitation is due to the studies available we were unable to examine whether properties (e.g. healthiness) or presentation of food determine the effect of portion size on daily energy intake and this may explain the observed heterogeneity. There was suggestive evidence of publication bias and some of the included studies scored high for markers of risk of bias. Analyses accounting for publication bias still resulted in a significant (but slightly smaller) effect of portion size on energy intake. Effect sizes were largely from adult studies and therefore may not be generalisable to children. The number of eligible studies was relatively small and therefore caution should be taken in the interpretation of some of the reported subgroup analyses. Studies also differed in some methodological features (e.g. compulsory eating of food *v.* not) but we found no evidence that results differed. Most studies were short in duration and measured energy intake for 1–2 d, therefore further studies examining the effect portion size on daily energy intake and body weight over longer time periods would be valuable. Studies did not tailor portion sizes provided to individual participant energy needs and this may have resulted in underestimation of the effect that portion size has on body weight. A further limitation is that the majority of studies were laboratory-based and therefore may not be reflective of real-world eating due to social desirability concerns^(45,46)^. A recent study found that the effect of portion size on short-term energy intake was larger when tested in the real world *v.* laboratory^(47)^, therefore we presume that the reliance on laboratory-based studies in the present meta-analysis would be more likely to under rather than overestimate the effect of portion size. A further consideration is that although test foods used in studies tended to be selected to be representative of the types of foods eaten by study populations (e.g. palatable and commonly consumed), it may be the case that among some participants foods consumed in everyday life are less palatable or energy dense and therefore larger portion sizes of such foods may exert a less pronounced increase to energy intake.

### Conclusions

Smaller food portion sizes decrease daily energy intake and there is evidence that over time this may result in lower body weight. Reducing food portion sizes may be an effective population-level strategy to reduce obesity.

## Supplementary material

For supplementary material accompanying this paper visit https://doi.org/10.1017/S0007114522000903. click here to view supplementary material
