# Supplementary figures and images for: Downsizing food: a systematic review and meta-analysis examining the effect of reducing served food portion sizes on daily energy intake and body weight
Source: Br J Nutr. 2022 Apr 7;129(5):888–903. doi: 10.1017/S0007114522000903 (PMC9975786; doi:10.1017/S0007114522000903)

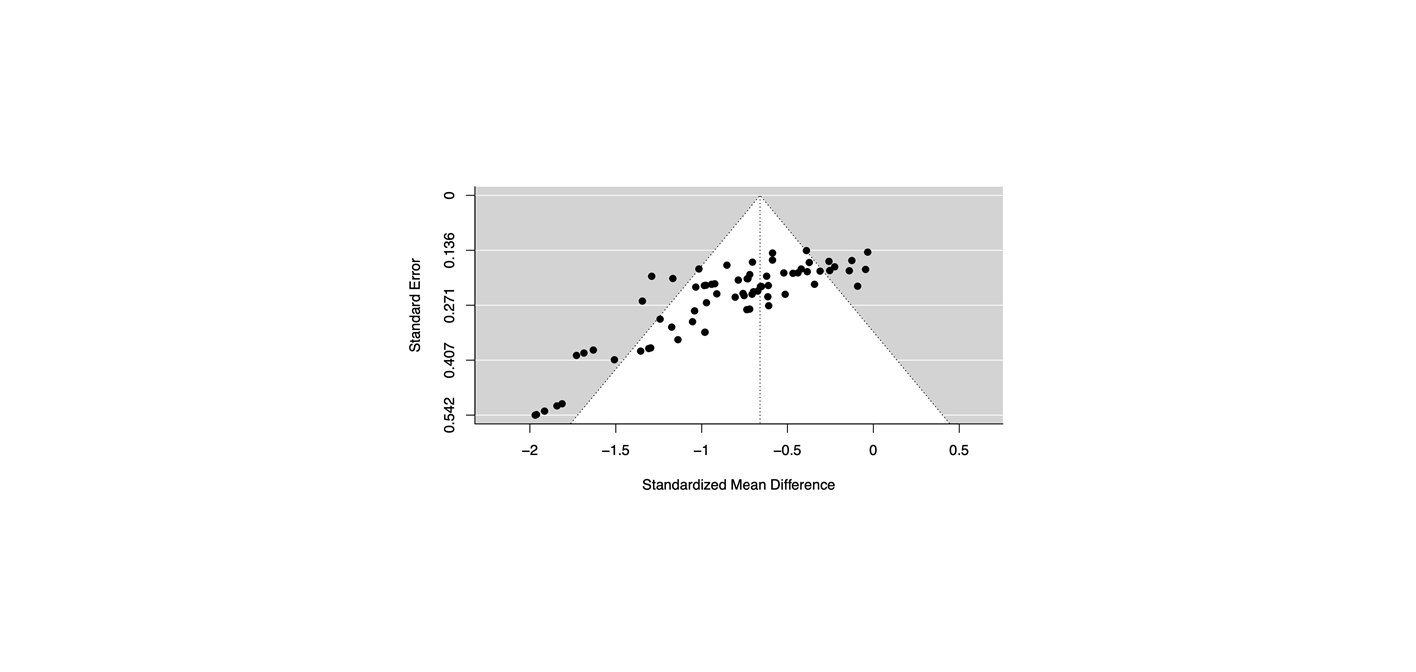

Supplement: Supplementary file 1 [file S0007114522000903sup.zip › S0007114522000903sup002.tiff]

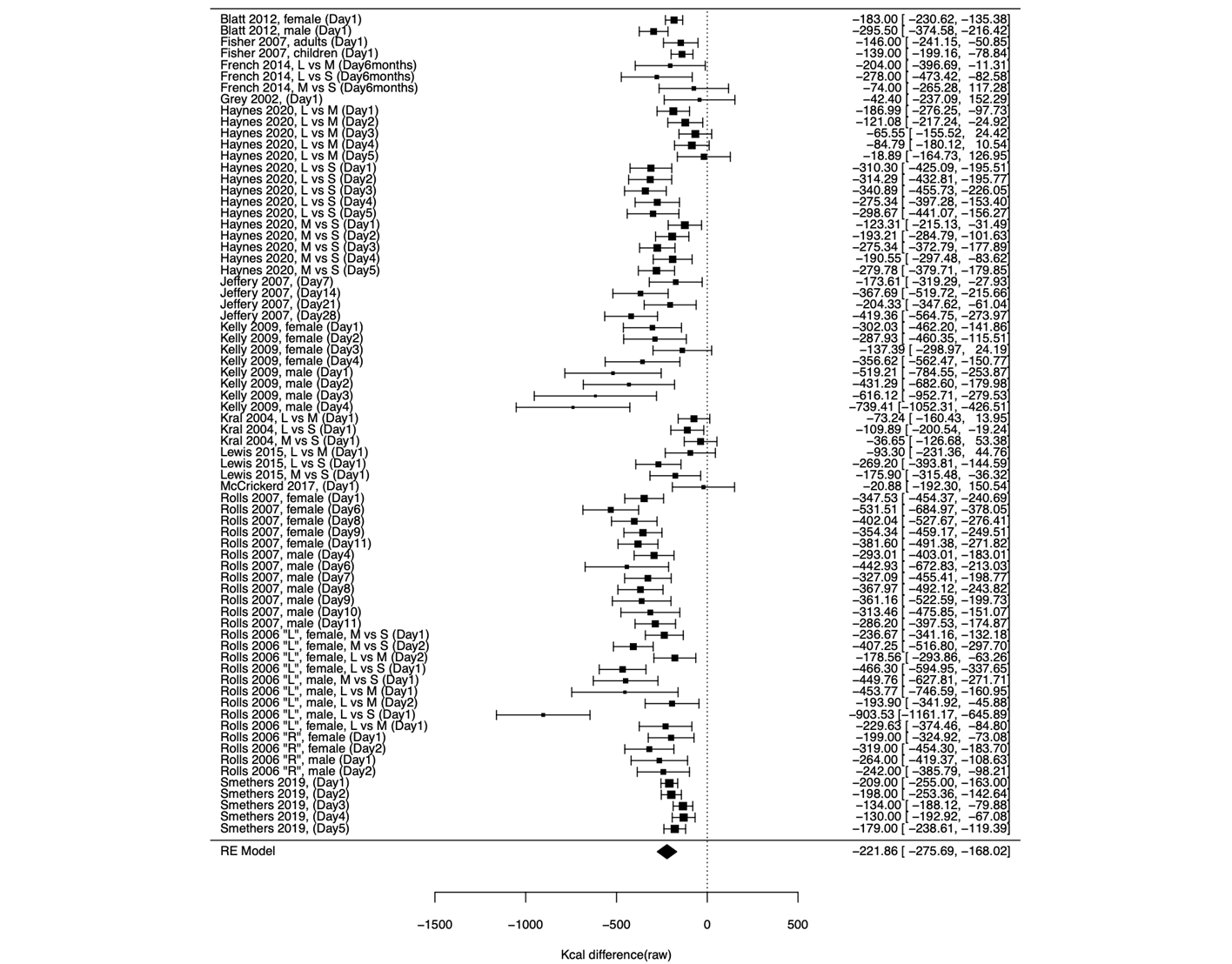

Supplement: Supplementary file 1 [file S0007114522000903sup.zip › S0007114522000903sup004.tiff]
